# Financing of non-communicable diseases in Afghanistan

**DOI:** 10.1186/s12939-025-02423-4

**Published:** 2025-04-09

**Authors:** Narges Neyazi, Ali Mohammad Mosadeghrad, Maryam Tajvar, Najibullah Safi

**Affiliations:** 1 International Campus, school of public health, Tehran University of Medical Sciences, Tehran, Iran; 2 Health system development department, World Health Organization, Kabul, Afghanistan; 3 Department of Health Management, Policy and Economics, School of Public Health, Tehran University of Medical Sciences, Tehran, Iran

**Keywords:** Financing, Non-Communicable diseases, Health system, Excise tax, Afghanistan

## Abstract

**Background:**

Afghanistan is suffering from a triple burden of diseases. One of every two Afghan is dying due to non-communicable diseases (NCDs). The national health account report shows that people are paying 77% of health expenditure from their pocket especially for diagnostic and treatment purposes. Considering the huge number of mortality and health expenditure related to NCDs, this paper aimed to analyze the financing system of NCDs and provide recommendations to the policy makers and program managers in national and international health institutions.

**Methods:**

A qualitative method was used to interview with 39 experts in health system of Afghanistan during 2019 to 2021. A self-developed interview guide was used for data collection. For analysis of data, we used deductive framework method and used the six building blocks of the health system as a framework, in this study for financing.

**Results:**

In analyzing the financing of NCDs in Afghanistan, the findings are summarized in four categories as below. The strength points are donor-funded packages of health services, producing the national health accounts reports, implementing minimum excise tax on tobacco or sugar sweetened beverages and the existence of some guiding policy documents such as revenue generation strategies. The weak points are low budget allocation to health by the government, centralized financing system in Afghanistan, lack of key NCDs indicators in health information system, and high out of pocket payments. On the other hand, the opportunities are high level of Out-of-Pocket payment, corrective tax on tobacco and sweetened beverages, availability of implementing NGOs in health sector. Also, threats are weak governance structure and risk of corruption in the health sector, lack of public trust on the government, barriers to implementing Public Private Partnership program, low health literacy of people, collusion between public and private sector, and long bureaucratic process.

**Conclusions:**

In general, health financing is closely linked to other health systems’ functions especially governance, health information management, health workforce management, provision of medicine, medical supplies, and technology. Thus, to have functional health financing, we need to consider these intercorrelations and provide synergies among the building blocks of health system.

## Background

Non-Communicable Diseases (NCDs) are responsible for nearly 74% of deaths worldwide [1]. The four main diseases are cardiovascular diseases (CVDs), cancers, diabetes, and Chronic Obstructive Pulmonary Diseases (COPD). The major unhealthy risk behaviors are physical inactivity, unhealthy diet, smoking, and harmful use of alcohol which lead to high biological/metabolic risk including overweight/obesity, high blood pressure, high blood glucose level and high levels of fat in the blood. NCDs impose a direct significant burden on economic and social development. During the period 2011–2030, NCDs will pose more than US$ 30 trillion which represents 48% of global Gross Domestic Product (GDP) in 2010. It will cause millions of people to fall below the poverty line. They lead to high treatment costs and cause a huge indirect economic lost through considerable productivity losses because of premature mortality, absenteeism, early labor force exits, and work at lowered capacity [2]. In 2020, 10.8% of global GDP was spent on health which was highly unequal across income groups [3]. 

Afghanistan is experiencing a triple burden of diseases where more than 50% of deaths were attributed to NCDs in 2019. This is predicted to increase to nearly 60% among women in 2030. In addition, nearly 70% of Years Lived with Disability will be due to NCDs till 2030 in this country [4]. The health expenditure in Afghanistan also had a 4.7% increase within one decade (2009–2019). However, the main driver was increasing Out Of Pocket Expenditures (OOPE). Afghanistan National Health Account (NHA) 2021 shows more than 77% of health expenditure is Out Of Pocket (OOP) payment. This report also shows NCDs including injuries are responsible for 20% of total health expenditure in 2021. In addition, 41% of health expenditures went to medical goods dispensed to outpatients [5]. In 2021, 16.8% of Afghanistan’s GDP was spent on health. However, the GDP in Afghanistan was 14.2 billion dollars in 2021 (very much less than its neighbors like Iran’s GDP (626.1 billion) and Pakistan’s GDP (374.7 billion)) [6]. 

NCDs and their major risk factors are not included in two packages of basic health services (BPHS) in primary care level and essential package of hospital services (EPHS) at secondary and tertiary level. So, as these two packages are funded by international donors such as the World Bank, there is no option for financing management of these diseases in the country, except paying from pocket by Afghan population to predominantly private sector service providers inside the country. In addition, Afghans are spending million dollars on diagnoses and treatment of their diseases specially NCDs in neighboring countries such as Iran, Pakistan, and India [7]. 

The six building blocks of a health system (service delivery, governance, financing, human resources, medicine and medical technology, health information system) are interdependent and work together to strengthen the health system. Health financing is closely related to the governance building block and together have a big impact on access to health care services [8]. Financing the health systems refers to three functions: the mobilization, accumulation, and allocation of money to cover the cost of health services to the people, community, and the health system. Parallel to these functions, the main concept of health system financing is that allows the people to use the health services without experiencing financial hardship or impoverishment [9]. Addressing the burden of non-communicable diseases through provision of quality health care needs a sustainable financing mechanism within the Afghan health system, for which no study so far considered it.

Taken into account that NCDs are not included in packages of health services (BPHS and EPHS) and there is no defined mechanism for financing them, we aimed to analyze the existing and potential financing mechanisms of NCDs in Afghanistan, highlighting the strengths, weaknesses, opportunities, threats and providing solutions. This analysis would help policy makers to invest, improve, or reform the existing mechanism for NCDs financing, and develop innovative and context-based financing strategies for NCDs in Afghanistan.

## Method

**Fig. 1:**
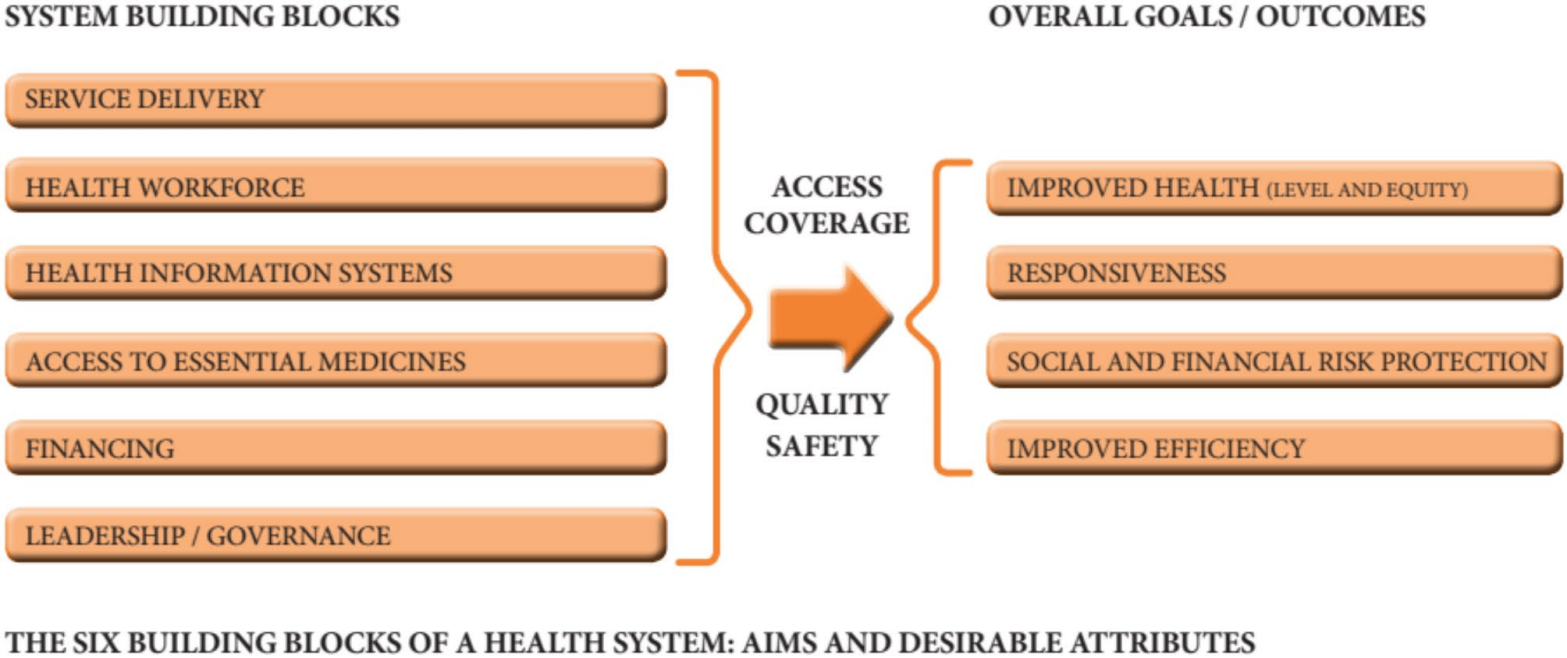
The six building blocks of health system: aims and desirable attributes. Source: WHO, monitoring the building blocks of health systems, 2010

We used a qualitative design - interpretive phenomenology method in this study [10]. Semi- structured interviews, as a most common method [11], were used to gain a full and deep understanding of existing situation of the strengths, weaknesses, opportunities, and challenges in the financing of NCDs currently in Afghanistan. As a deductive framework analysis method, we applied a six-building block framework of health system which was developed by WHO in 2010 [10, 12]. We only report the findings for the financing building block in this paper (Fig. 1).

A questionnaire was developed based on the literature review and health system building blocks. We conducted three pilot interviews to increase the interviewer’s skills in communicating with experts and add to the validity of the research. We interviewed 29 experts from 2019 to 2020. After regime change in August 2021, radical changes especially in financing health system have happened in Afghanistan. In early months, donors froze their funds and then they channeled their financial support through UNICEF and WHO to the out-contracted NGOs. These NGOs are responsible for health service delivery at facility level. To gain full insight into the effect of this political change, we did additional 10 interviews during 2021.We applied the purposive snowball sampling method with maximum variation among experts. The participants included practitioners, program managers, policy makers, different service providers, and researchers. We sent a summary of the interview guide to the participants for their consideration prior to the interview. The interviews were face to face in the experts’ workplace or virtually through the telephone/ Skype/ WhatsApp conversation. The average time of interviews was 12 min (minimum was 8 min and the maximum time was 17 min). We stopped interviewing the experts when we reached an information saturation level. We recorded the interviews and transcribed them for increased accuracy after each interview. The interviews were in local language, and they were translated to English. To ensure the quality of transcriptions, we conducted a back translation.

Most of the interviewees were male (92.6%), with master’s degree (85.2%), between 30 and 40 years old (51.9%) and with 5 to 10 years of experience (59.3%) and most of them worked as policy maker (40.7%) and service provider (37.0%). More detail about all categories of participant can be found in our previous publication about governance of NCDs in Afghanistan [12]. 

The framework method includes transcription, familiarization with interview, coding, developing a working analytical framework, applying the analytical framework, charting data into the framework matrix, and interpreting the data [11]. All recorded interviews were transcribed verbatim in full. The interview transcripts were read several times, and initial codes were generated. Similar codes were grouped into sub- themes and then into themes. At the end, considering the research objective, the narrative was reported in terms of a purposive story. All the quotes are presented by “p” in the result section.

Participation in the study was voluntary, and they could stop participating in the study at any point felt uncomfortable. We obtained the consent of participants regarding inclusion and audio taping too. Also, all the gathered data was confidential, and the personal perspective of researchers were not involved in the phases of data collection, analysis, and reporting.

## Result

Financing is one of the six building blocks of health system with three sub-themes. The sub-themes are financial resource collection, financial resource pooling, and health service purchasing mechanism. We present the view of experts about each sub-theme in this section (Table 1).

**Table 1:** Sub- themes and codes for analysis of financing of the NCD management system in Afghanistan

	Strengths	Weaknesses	Opportunities	Threats	Solutions
Revenue collection	Funded two packages of BPHS and EPHS; amendment of law toward financing the health system; implementation of some funded programs for NCDs such as cancer center and heart diseases center in Indra Gandi hospital; generation of National Health Account reports and open market of Afghanistan	Under-funded health system; low government budget allocation to health sector; lack of financing mechanism for NCDs; and centralized financing system through the MoF	High level of OOP; collection of corrective tax;	Extensive corruption; lack of experts for advocacy; donor priority on mother and child and communicable diseases; changing the government regime; brain drain; competition for available resources among different ministries	Allocation of corrective tax to the Ministry of Public Health; increasing the GDP percentage to the health sector; establishment of independent income account for specific health sector organizations; integration of NCD services into the donors’ packages; training the health economics specialist; amendment of current laws for better financing; increasing the transparency and accountability; conducting the gap analysis and assessments in health financing system
Fund pooling	The feasibility study for implementation of social health insurance; independent income source for the municipality; development of NHA 2017 based on the diseases	Low public health expenditure and high OOP; lack of electronic system; lack of social insurance; insufficient health per capita; No good coordination for fund raising and fund pooling.		Lack of public trust on government; lots of barriers for implementing PPP; extensive corruption; freezing the financial resources of the country after regime change	Starting the social health insurance for government hired officers; establishment of a group of researchers from different countries; integrate the off budget with on budget; sending more staff for getting MSc in health financing; making the financial system digitalized; capacity building of HEFD; increase the advocacy for NCDs; establishment of charity collection system; promote the inter-sectorial coordination
Purchasing/ provision of services	Starting PPP program in health sector; receiving fund from donors; transparent service procurement system in the region	Donor dependent health system; insufficient budget for BPHS and EPHS; lack private sector regulation system; lack of a proper resource use and distribution system; lack of required infrastructures for NCDs	Available experienced NGOs; working on development of DPT3 and IPEHS; donors who support research	Collusion between public and private sector; low health literacy of people; long bureaucratic process; weak monitoring of medicine market; lack of knowledge about health insurance; lack of a good model to address NCDs and intervention of politicians in health programs	Budget allocation based on population age and need; strengthening the monitoring system; regulation of private sector in terms of small-scale PPP; allocation of specific budget to research; establishment of national health insurance system; development of tertiary care package and establishment of result-based financing mechanism in health sector

### Revenue collection

In this subfunction, we explored that the strength points are existing of donor-funded two packages of BPHS and EPHS; amendment of law toward financing the health system; implementation of some funded program for NCDs such as cancer center and heart diseases center in Indra Gandi hospital; generation of National Health Account reports by Health Economic and Financing Department (HEFD) in MoPH; and open market of Afghanistan. A director general from a national research and public health institute said, *‘fortunately*,* the law article which has closed our hands*,* was amended. The previous law obliged the government to provide the health services free of cost for all public*,* while this led to increase payments under the tables along with other kind of illegal payments. After this amendment*,* only primary healthcare is free of cost and the public should pay for other services which can increase the income for the health sector’ (p. 5).* In other hand, a public health expert from WHO in Afghanistan said, *‘at least*,* we have donor fund for two existing packages of BPHS and EPHS’ (p. 11).* In addition, a previous director for grant management of MoPH said, *‘prevention and treatment of some NCDs and their risk factors are included in the BPHS and EPHS which are funded by donors’ (p. 17).* A marketing manager from a private medicine company said, *‘there is an open market for medicine in Afghanistan which is regulated based on the demand and supply and the affordability of people to purchase the medicine. The government has no right to regulation of prices in an open market and only can develop the policies’ (p. 13).*

The weak points are the under-funded health system, low government budget allocation to health sector; lack of financing mechanism for NCDs and centralized financing system through the MoF. A senior official from HEFD department of MoPH said, *‘the NCD department does not have enough capacity for fundraising*,* for which I*,* as a health financing officer*,* did not hear anything about their advocacy for fund raising’ (p. 26).* Most governmental organizations have no sufficient budget for their activities and despite having an income source, they don’t have independent bank accounts to receive their income and spend it for their programs. A senior official from the National Environmental Protection Agency said, *‘we don’t have source of income for our organization and all the collected fees go to the ministry of Finance’ (p. 3).* A senior manager from NHMRA also said, *‘we don’t have an independent income collection system in our organization and our collected fee goes to the MoF*,* so we do not have sufficient budget for our workshops*,* seminars and even salary of our staff’ (p. 15).*

The opportunities are high level of OOP (73–75%); collection of corrective tax from tobacco and sugar sweetened beverage and other harmful food traders; existing of data especially on mortality burden; and existing some donors. A senior officer from HEFD of MoPH and a director of a NGO in Kabul emphasized, *‘the high level of OOP is an opportunity which can be managed by the government and be used to develop an insurance system in Afghanistan’ (p*,* 20; p. 21).*

The threats are the risks of corruption in the health sector which prevent stakeholders from using the opportunities, for example there is no commitment to allocate the corrective tax to the health sector by MoF. In the other hand, continuous unstable political situation throughout the country affects the government commitment to the health sector, which means the health sector is not the priority of Afghanistan. A senior public health expert said, ‘*a witness told me that a cigarette importing company were inviting the cabinet of previous government every week or gave the money to the ministry of public health. The system is very corrupt’ (p. 32).* A senior manager from the health promotion department said, *‘most of the fund are secured by the donors for health service delivery*,* unfortunately*,* we don’t have expert for advocacy among the donors*,* the one who knows strategy’ (p. 18).* Many interviewees pointed that the priority of donors in Afghanistan are the mother and child health programs and communicable diseases control, because a long time needs for return on investment for NCDs and it is not prioritized by donors. Moreover, a senior manager from health promotion department said, *‘most of Afghan patients travel to India and Pakistan for receiving diagnosis and treatment services for their diseases which withdraw the financial resource for health sector from Afghanistan’ (p. 4).* Competition for available resources among different ministries, changing the government regime, personal interest of leaders in policy making and planning and brain drain are other threats for this area.

For improving the revenue collection sub-function, the interviewees suggested that the corrective tax on harmful food should be allocated to the Ministry of Public Health directly, in addition the government should increase the GDP percentage to the health sector and allow the organizations to have independent income account. A senior official from the National Environmental Protection Agency said, *‘we don’t have even financial dependency in our universities*,* all governmental organizations receive their annual budget from MoF*,* for example all our income from giving certificate to the other organizations or from environments protection programs go to the MoF*,* I think we should have an independent account to collect our income directly and use it for our institutional development’ (p. 3).* Integration of NCDs services into already existed packages by donors and investing in training health economics specialists are other solutions. The officials from health promotion department also proposed, *‘donors should integrate the NCDs related health promotion services in their packages for example in their vaccination or nutrition program. Moreover*,* each ministry should allocate 2–5% of its budget to health promotion’ (p. 9*,* p. 18).* Other experts in this study suggested that the MoPH should invest in the training of economic specialists and develop the related policies on fee collection and spending system. Almost all the interviewees emphasized that the government should work toward providing a sustainable security in Afghanistan, the economic situation should be improved by increasing the GDP, and then the investment in the health sector should be promoted. They also emphasized that an advocacy strategic plan should be developed for government and donors. They mentioned that the amendment of current laws toward better financing of the health sector is essential, and attention should be paid by the government leadership. A senior advisor from HEFD said, *‘now after development of NCD strategy*,* a financial gap analysis should be conducted and the resources and interventions should be prioritized*,* moreover*,* we should increase the transparency in the public health system through dissemination of our activities’ report to the civil society and different association’ (p. 29).* A senior public health expert from an international organization said, *‘an assessment should be conducted on the increment of health per capita and based on the WHO recommendations*,* a line should be added to the budget expenditure specifically for NCDs’ (p. 32).* Another expert from WHO said, *‘the NCD should be put in the top agenda in meetings with the donors*,* moreover*,* one focal point should be allocated to each NCDs in the related department for better advocacy and coordination. The NCD department should have prepared proposals for separate donors*,* it can get help from private hospitals and medicine companies to finance its programs too. WHO can assist this department in developing the concept notes’ (p. 36).*

### Fund pooling

The strength points are conduction of feasibility study for implementation of social health insurance by MoPH; independent account for municipality; the national health account reports for 2017 and afterwards which were developed based on the diseases and now there is information on how much is spending on NCDs in the country (25% of the health expenditure).

The weak points are low public health expenditure per capita (3.5 dollar) and high out of pocket payment (OOP) (75%); and low investment on NCD primary care. Most of these out-of-pocket payment goes to seeking healthcare services in other countries mostly India and Pakistan for diagnosis and treatment of advanced non-communicable diseases like cardiovascular diseases, diabetes and cancers.A senior health promotion manager said, *‘most of the GDP percent allocated to the health sector spent on the hospital establishment and hospital services and the very limited budget is allocated to health promotion’ (p. 4).* Lack of insurance system in Afghanistan is another huge gap for financing of NCDs. A cardiac surgeon from a private hospital said, *‘most governments in the world finance primary care and even the donors*,* it is the case of other countries but with this difference that in other country*,* there is social insurance for secondary and tertiary health care’ (p. 22).* In addition, the interviewees mentioned that there is no good coordination for fund raising and fund pooling in the health sector, especially for NCDs.

The threats are lack of public trust on the government; lots of barriers for implementing Public Private Partnership (PPP); extensive risk of corruption; and freezing the financial resources of the country after regime change. An M&E senior advisor in MoPH said, *‘people do not trust on the government due to unstable political and economic situation in the country*,* so they don’t have willing to establish the social health insurance system’ (p. 8).* He also added that, *‘there are a lot of administrative and procurement barriers in front of implementing PPP*,* besides*,* most of donors’ financial assistance goes to abroad again due to the recruitment of international consultants*,* admin staff and administrative cost’ (p. 8).* The other factors include existing laws which prevent the MoPH from implementing the revenue generation strategy, extensive corruption in the health system and lack of digitalized system for health financing. A senior public health expert from an international organization said, *‘we had a good resource pooling and resource sharing over the past 20 years*,* many donors pooled their resources to fund the health system of Afghanistan*,* but right now all the financial resources of Afghanistan has been frozen*,* in this situation if we can maintain our past achievements is also good’ (p. 32).*

The suggested solutions are increasing the public awareness about the social health insurance system and starting the social health insurance for government hired officers at first stage; integration of off budget with on budget, so MoPH can spend the budget based on its priority areas such as NCDs; establishment of independent account for collection of income for different organizations in the health sector; promote the inter-sectorial coordination between other ministries such as Ministry of Finance (MoF), Ministry of Education (MoE), Ministry of Public Health (MoPH), Ministry of Higher Education (MoHE) for NCDs; and advocacy for a new and integrated package of health care. The director general of a national research institute said, *‘a group of researchers should be established from different regions of the countries to design the fee collection and spending system in Afghanistan hospitals’ (p. 5).* A senior researcher from an international NGO in Kabul also said, *‘the MoPH should invest in the capacity building of staff to have better financing system and should train the health financing or health economics specialist’ (p.1).* A public health expert from UNICEF also emphasized, *‘more staff should be sent to take their master’s degree and be trained in health economic areas’ (p. 14)*. Other experts interviewed in this study suggested that MoPH should improve the debate and discussion among different sectors and determine the problem and NCDs burden for further advocacy; a charity collection system should be established, and the security of country should be guaranteed for a sustainable charity collection system. A senior public health expert from an international organization also suggested, ‘*after de-freezing our financial resources by international organization*,* we should conduct an assessment to know how much of our resources should be allocated to NCDs*,* establishment of shared fund in private sector to finance the private hospital is another option but require transparency and trust’ (p. 32).*

### Purchasing/provision of services

One of strength point is starting the PPP program in the health sector. A senior cardiac surgeon from a private hospital said, ‘*I have heard that two national hospitals of Sheikh Zahed and Jenah are going to be outsourced*,* so the work on the Private Public Partnership is in progress’ (p. 22).* Other strength point is development of good national health financing documents such as guideline for budget expenditure for hospitals; the four-round published NHA which have been used for sensitizing the MoPH leadership toward NCDs; existing well developed revenue generation strategy for 2018–2023 including the tax on tobacco or sugar sweetened beverage. A senior official from HEFD said, *‘we worked with hospitals in Kabul city*,* we estimated the NCDs cost in hospitals of Kabul and have data about the actual budget needed for NCDs and the current allocated budget and can determine the gap between these two indicators’ (p. 26).*

The weak points are donor dependent health system; high OOP; insufficient budget for BPHS and EPHS to cover NCDs. The head of oncology department from one of national hospitals said, *‘the allocated budget to our center is not sufficient and the patients have to purchase their chemotherapy medicines themselves’ (p. 6).* Another weak point is the lack of a proper regulation system for the private sector. A senior marketing manager from a medicine company said, *‘the currency price of Rial*,* Dollar or Ropia has its effect on the medicine price*,* so we can find a medicine with different prices in the market’ (p. 13).* The interviewees also pointed that there is no good marketing system in the health sector of Afghanistan. The focus of international partners is on financing of mother and child health programs and there is no capacity in MoPH for planning and prioritizing the problems. The health promotion manager from MoPH said, *‘our international partner in health promotion focuses on mother and child health and they don’t allocate the budget to control and prevention of NCDs including conduction of the research. In addition*,* we don’t have any on– budget allocation to our department*,* and there is not any cost effectiveness analysis for interventions of our department’ (p. 18).* A senior manager from Afghanistan National Public Health Institute also said, *‘the MoF and donors don’t invest properly on NCDs*,* and the people also go to hospitals at the end stage of the diseases*,* and they don’t have willing to invest on the preventive measures for NCDs’ (p. 20).* In addition, the director of a local NGO said, *‘75% of health expenditure is paid by people as OOP*,* and they pay for those services in health facilities where they don’t have the required infrastructure to handle those chronic conditions (NCDs) in Afghanistan. So*,* people do not receive the desired result from their treatment*,* we don’t have medicine and equipment which are required for NCDs*,* on the other hand*,* the private sector also can provide NCD services to the extent that government allows them’ (p. 21).*

The opportunities are available good multiple NGOs working in the health sector and existing the donors who support the research.

The threats are collusion between public and private sector; low health literacy of people; long bureaucratic process; lack of knowledge about health insurance; lack of a good model to address NCDs; and intervention of politicians in health programs. A senior researcher from an international NGO in Kabul said, *‘40–50% of OOP is spent on medicine procurement and there is no control on medicine price and quality’ (p. 1).* In addition, a general director of a national research institute said, *‘the collusion between public and private sector for referring the patients leads to high OOP’ (p. 5).* A senior official from the M&E HIS department said, *‘intervention of politician and local powerful individuals who intervene into the government’s program are the big threats for proper financing and governing the health system in Afghanistan’ (p. 10).* A director from an NGO said, *‘the government does not have any model for NCDs*,* so private sector also follows the government model*,* just the property is private. So*,* the private sector does not have structure or professional staff*,* in addition*,* most of staff and doctors who work in private sector also work in public hospitals and they have limited knowledge as much as the government provides the opportunity to learn for them’ (p. 21).*

The proposed solutions are budget allocation based on population age and need; strengthening the monitoring system; regulation of private sector in terms of small-scale PPP; allocation of specific budget to research; establishment of national health insurance system; development of tertiary care package and establishment of result-based financing mechanism in health sector. A senior health promotion manager from MoPH said, ‘*the MoPH should allocate the budget to the different department considering the population age and their needs. In addition*,* the MoPH should create a coordination system for NGOs to align their programs toward MoPH goals and objectives*,* so we will have more synergy toward collecting and spending the financial resources with a better outcome’ (p. 4).* A general director from a national public health institute also said, *‘the MoPH should strengthen the control and monitoring throughout the health system*,* in addition*,* a series of research studies should be conducted to find out about the root causes of financial problems and then the corrective actions should be taken’ (p. 5).* An M&E senior Advisor from MoPH said, *‘we should get out from this financial situation*,* we should use the health insurance*,* user fee*,* pay for performance and provision of health services in two or three shifts in national hospitals’ (p. 8).* On the other hand, one of the director generals of MoPH said, *‘the government should plan in a multi- dimensional approach considering security*,* education*,* economic and health development’ (p.10).* One of public health experts from UNFPA also said, *‘we need strategic purchasing in which the government purchases secondary and tertiary health care from the private sector’ (p. 19).*

## Discussion

In this paper, we highlighted the strengths, weaknesses, opportunities, and threats in financing system of NCDs management in Afghanistan. In addition, we also provided the solutions.

Existing supportive laws are both an opportunity and a threat. Although, according to the law, only primary health care is free to the public, the collected resources from secondary and tertiary care provision are not allocated to the health sector, MoPH and health related programs. This led to scarcity of resources for financing the health programs especially for NCDs. Resource generation for management of NCDs including prevention, diagnosis, treatment, and rehabilitation needs legal support nationally and internationally. WHO Framework on Convention on Tobacco Control (WHO FCTC) is an example of international legal framework to control the tobacco use and implementing the restriction on its marketing. Afghanistan also signed this convention, and in 2015 enacted its first anti-tobacco law which includes extensive ban on smoking in all places [13]. The excessive tax on tobacco products, sweetened beverage and alcohol should be increased in line with inflation and GDP growth in Afghanistan and ideally, the resulted revenue should be earmarked for health spending purposes [14]. Sin tax is a source for sustainable health financing in many countries like Thailand, England, Australia, Philippines, South Africa, and Vietnam [15]. Some of strategies to have sustainable health financing system are (1) increasing the health contribution of the GDP and government budget; (2) six tax for unhealthy foods, sweetened beverage, and drugs; (3) Tobacco tax; (4) Value added tax (VAT) and allocating a percentage of VAT as a health tax; and pollution charges [16]. Afghanistan also should consider these strategies for sustainable health financing with a focus on NCDs.

The existing frameworks and regulations to initiate the PPP programs also are ineffective. Based on India experience, which is one of successful countries with PPP models, there is a need for well-enunciated governance framework and policy, detailed assessment of market behavior and regulatory mechanism, and building the organizational structures [17]. 

A strength pointed out by many experts is two packages of BPHS and EPHS. These two packages are funded by international donors, mainly the World Bank, and were first established in 2005. However, these packages were developed for epidemiology and demography of that time with a focus on maternal and child health and communicable diseases. If the diagnosis and treatment of NCDs and their risk factors become included in these two packages, so the health care needed for NCDs and their risk factors will be financed by donors in public health facilities in primary, secondary and tertiary health care. According to experts in this study, there are many private health facilities which provide secondary and tertiary care for diagnosis and treatment of patients with NCDs. However, they do not have the required standard infrastructure, professional staff, and medical technologies for these diseases. 27% of 63,723 trained health workers practice in the private sector. These private health facilities including complex centers, hospitals, OPD clinics, diagnostic centers, and laboratories are not regulated, monitored, and supervised by MoPH and other organizations such as Afghanistan Medical Council (AMC), and Afghanistan Nursing and Midwifery Council (ANMC) [18]. So, all these lead to Afghanistan has the highest OOP in the region [19]. A systematic review identified the main drivers of OOP among patients with chronic diseases which are hospitalization and medications that lead to lost income, financial catastrophe, and foregone education [20]. Some strategies to reduce OOPs are mobilizing OOP payments on a pre-paid basis through formal or community-based risk pooling schemes; exemption process of fees for the poor, disabled, and disadvantaged; basic insurance scheme (retired workers are exempt from premium contributors, and the cost of their contributions is to be borne by their former employers; implementing diagnosis-related group (DRG)-based payment system and performance-based payment; developing clinical guidelines to control unnecessary diagnosis and treatment services [21]. 

Establishing HEFD within MoPH is another strength point. This department, with financial and technical assistance of WHO, produces National Health Accounts annually. This report provides very strong evidence for decision making and program planning in the country. However, due to recent political changes, many health economic experts of this department left the country. Many experts in this study strongly recommended training in health economics within the country, so they can maintain the established and developed system. Establishment of multisectoral coordination mechanisms also would result in a synergy between the health-related programs of different ministries. The “Health in All Policies (HiAP) concept” should be introduced widely at government level and the government leaders should support it strongly [12]. NCD Alliance recommended to reduce OOP, countries can promote multisectoral partnerships to optimize efficiency in health systems and care delivery; engage people living with NCDs and civil society; prioritize development aid that supports sustainable domestic resource mobilization [22]. 

Experts also pointed out that there is an information asymmetry between the patients and service providers, mostly clinicians. This results in prescribing unrequired and unnecessary diagnostic tests and medicines which leads to catastrophic out of pocket payments, especially in the private sector. To prevent it, strategic purchasing was recommended. Thailand and Indonesia are two countries with strategic purchasing system that could decrease the OOP payments [23]. According to WHO, “*purchasing refers to the allocation of pooled funds to healthcare providers for the delivery of health services on behalf of certain groups or the entire population*”. We can call the purchasing is strategic, when the allocations are linked to the health needs of the populations and to the performance of the service providers, with a focus on the efficiency gains, managing expenditure growth and equitable distribution of resources [24]. Strategic purchasing also contribute to Universal Health Coverage (UHC) [25] and to a resilient health system [26]. In current modality, we have strategic purchasing for BPHS and EPHS, however, the government and the international stakeholders should invest on establishing a mechanism for strategic purchasing from private sector. This will lead to increase the efficiency [27] and decrease the OOP significantly, while the quality of services will be improved.

This study has some limitations. First, the regime change in Aug 2021 and subsequent radical changes in the health financing system in Afghanistan might have impacted the study results. The additional 10 interviews conducted in 2021 may not fully capture the evolving situation. In addition, using purposive snowball sampling may introduce selection bias as participants are likely to recommend others with similar perspectives, potentially limiting the diversity of views.

## Conclusion

In conclusion, the management of non-communicable diseases (NCDs) in Afghanistan faces a complex array of strengths, weaknesses, opportunities, and threats within its financing system. Key challenges include the misallocation of resources collected from secondary and tertiary care, leading to a scarcity of funds for NCD programs. Legal frameworks at both national and international levels, such as the WHO Framework on Tobacco Control (WHO FCTC), offer opportunities for resource generation through taxes on tobacco, sweetened beverages, and alcohol. However, there is a need for increased allocation of these revenues to health spending.

Strategic recommendations include amending national laws to support public-private partnerships (PPPs), integrating NCD diagnosis and treatment into the BPHS and EPHS packages funded by international donors, and enhancing the regulation and monitoring of private health facilities. Additionally, reducing out-of-pocket (OOP) payments through risk pooling schemes, exemptions for disadvantaged groups, and strategic purchasing are crucial steps toward achieving sustainable health financing.

The establishment of the Health Economics and Financing Directorate (HEFD) within the Ministry of Public Health (MoPH) and the production of National Health Accounts are strengths that provide robust evidence for decision-making. However, political changes have led to a loss of expertise, emphasizing the need for training in health economics. Multisectoral coordination and the “Health in All Policies” (HiAP) concept should be promoted to enhance efficiency and support sustainable domestic resource mobilization.

Addressing information asymmetry between patients and service providers through strategic purchasing, as demonstrated in Thailand and Indonesia, could significantly reduce OOP payments and improve the quality of healthcare services. These measures will contribute to Universal Health Coverage (UHC) and a resilient health system, ultimately improving the management of NCDs in Afghanistan.

## Data Availability

The interview transcribes generated and analyzed during the current study are not publicly available as the informed consent applied only for the use by the research team. If desired, the data can be viewed and reviewed together with the corresponding author.

## Abbreviations

NCDs: Non-communicable diseases
CVDs: Cardiovascular diseases
COPD: Chronic Obstructive Pulmonary Diseases
GDP: Gross Domestic Product
OOPE: Out Of Pocket Expenditures
OOP: Out Of Pocket
NHA: National Health Account
HEFD: Health Economic and Financing Department
MoPH: Ministry of Public Health
WHO: World Health Organization
BPHS: Basic Package of Health Services
EPHS: Essential Package of Hospital Services
NHMRA: National Health and Medicine Regulatory Agency
MoF: Ministry of Finance
NGO: Non-government organization
MoE: Ministry of Education
MoHE: Ministry of Higher Education
UNICEF: United Nations International Children’s Emergency Fund
M&E HIS: Monitory and Evaluation, Health Information System
UNFPA: United Nations Population Fund
FCTC: Framework on Convention on Tobacco Control
AMC: Afghanistan Medical Council
ANMC: Afghanistan Nursing and Midwifery Council
HiAP: Health in All Policies
UHC: Universal Health Coverage
